# Supplementary material for: Mechanisms governing the pioneering and redistribution capabilities of the non-classical pioneer PU.1
Source: Nat Commun. 2020 Jan 21;11:402. doi: 10.1038/s41467-019-13960-2 (PMC6972792; doi:10.1038/s41467-019-13960-2)
Supplement: Supplementary file 7 — Source data [file 41467_2019_13960_MOESM7_ESM.zip › Source_Data/FigureS3/FigureS3J_MotifScanOutput/homerResults/motif6.similar.html]

motif6

## Information for motif6

C
G
T
A
T
A
C
G
G
C
T
A
C
G
A
T
T
A
G
C
G
C
T
A
T
C
G
A
C
G
T
A
T
A
C
G
T
A
C
G
A
T
C
G
  
Reverse Opposite:  

T
A
G
C
A
T
G
C
A
T
G
C
G
C
A
T
A
G
C
T
C
G
A
T
A
T
C
G
G
C
T
A
C
G
A
T
A
T
G
C
G
C
A
T
  

|  |  |
| --- | --- |
| p-value: | 1e-158 |
| log p-value: | -3.649e+02 |
| Information Content per bp: | 1.524 |
| Number of Target Sequences with motif | 2043.0 |
| Percentage of Target Sequences with motif | 15.48% |
| Number of Background Sequences with motif | 2612.6 |
| Percentage of Background Sequences with motif | 7.16% |
| Average Position of motif in Targets | 406.0 +/- 331.4bp |
| Average Position of motif in Background | 321.4 +/- 195.5bp |
| Strand Bias (log2 ratio + to - strand density) | 0.0 |
| Multiplicity (# of sites on avg that occur together) | 1.09 |
| Motif File: | file (matrix) reverse opposite |

### Similar de novo motifs found

|  |  |  |  |  |  |  |  |
| --- | --- | --- | --- | --- | --- | --- | --- |
| Rank | Match Score | Redundant Motif | P-value | log P-value | % of Targets | % of Background | Motif file |
| 1 | 0.882 | C G T A T A C G C G T A T A C G G C T A C G A T T A G C G T C A T C G A C G T A T C A G T C A G | 1e-117 | -271.426179 | 28.29% | 18.49% | motif file (matrix) |
| 2 | 0.779 | C G T A C T A G A T G C G A T C G A C T A G C T C A G T A T C G G C T A C A G T A T G C G A C T A T G C | 1e-60 | -139.467074 | 12.18% | 7.33% | motif file (matrix) |
| 3 | 0.855 | G A C T C G A T C G A T A T G C C G T A C G T A C G T A A C T G | 1e-55 | -128.855981 | 18.75% | 12.96% | motif file (matrix) |
| 4 | 0.813 | G T A C A C G T A C G T A C G T A G T C C G T A C G A T A T G C | 1e-31 | -71.461100 | 10.78% | 7.43% | motif file (matrix) |
